# Forest-Based Networks

**DOI:** 10.1007/s11538-022-01081-9

**Published:** 2022-09-15

**Authors:** K. T. Huber, V. Moulton, G. E. Scholz

**Affiliations:** 1 University of East Anglia, Norwich, UK; 2 Bioinformatics Group, Department of Computer Science, Interdisciplinary Center for Bioinformatics, Leipzig University, Leipzig, Germany

**Keywords:** Phylogenetic network, Lateral gene transfer, Forest-based network, Tree-based network

## Abstract

In evolutionary studies, it is common to use phylogenetic trees to represent the evolutionary history of a set of species. However, in case the transfer of genes or other genetic information between the species or their ancestors has occurred in the past, a tree may not provide a complete picture of their history. In such cases, *tree-based phylogenetic networks* can provide a useful, more refined representation of the species’ evolution. Such a network is essentially a phylogenetic tree with some arcs added between the tree’s edges so as to represent reticulate events such as gene transfer, hybridization and recombination. Even so, this model does not permit the direct representation of evolutionary scenarios where reticulate events have taken place between different subfamilies or lineages of species. To represent such scenarios, in this paper we introduce the notion of a *forest-based network*, that is, a collection of leaf-disjoint phylogenetic trees on a set of species with arcs added between the edges of distinct trees within the collection. Forest-based networks include the recently introduced class of *overlaid species forests* which can be used to model *introgression*. As we shall see, even though the definition of forest-based networks is closely related to that of tree-based networks, they lead to new mathematical theory which complements that of tree-based networks. As well as studying the relationship of forest-based networks with other classes of phylogenetic networks, such as tree-child networks and universal tree-based networks, we present some characterizations of some special classes of forest-based networks. We expect that our results will be useful for developing new models and algorithms to understand reticulate evolution, such as introgression and gene transfer between species.

## Introduction

In evolutionary biology, it is common to represent the evolution of a set of present-day species using a *phylogenetic tree*, that is a rooted, graph-theoretical tree whose leaves correspond to the species Steel (2016). In recent years however, it has become increasingly recognized that phylogenetic trees may not provide an adequate means to represent the evolution of set of species in case the species or their ancestors have transferred or shared genetic material between one another in the past. This type of evolution is sometimes called *reticulate evolution*, and it includes evolutionary processes such as introgression, gene transfer, hybridization and recombination. Phylogenetic trees are not able to fully represent this type of evolution since they can only represent speciation or branching events [see, e.g., Huson et al. (2010), Chapter 4], and reticulate events require a graph where ancestors come together.

**Fig. 1:**
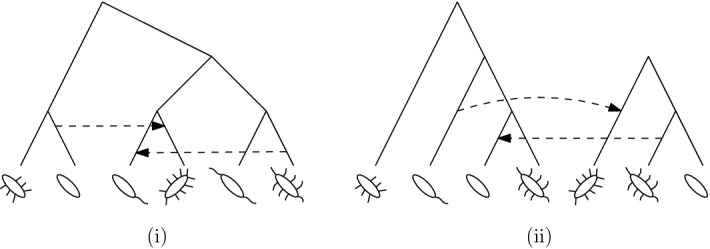
(i) A tree-based network and (ii) a forest-based network for a collection of bacteria. The dashed arrows indicate a lateral gene transfer event between bacterial ancestors

Despite this issue, phylogenetic trees can still be used as a starting point to represent reticulate evolution by, for example, taking some phylogenetic tree and then adding in extra edges to represent reticulate events [see, e.g., Makarenkov (2001)]. We illustrate this in Fig. 1i, where we have started with a base-tree representing the evolution of a hypothetical collection of bacteria, and added in some dashed arcs between arcs in the tree so as to represent past events where genes have been laterally transferred between ancestral species [see, e.g., Kunin et al. (2005) and Makarenkov et al. (2021) for some real-world examples in bacteria and viruses, respectively]. Mathematically speaking, the resulting graph-theoretical structure is an example of a *phylogenetic network*, that is, a rooted, directed acyclic graph with leaf-set corresponding to the present-day species [see, e.g., Steel (2016)]. Note that directed cycles are not allowed in such networks since, for example, a species cannot be an ancestor of itself.

Phylogenetic networks that are created by adding in edges to a phylogenetic tree to form a network are called *tree-based networks* Francis and Steel (2015). Since their formal introduction in Francis and Steel (2015), tree-based networks have created a lot of interest in the literature. For example, it is known that not every phylogenetic network is tree-based Iersel (2013), and as a result several elegant characterizations of tree-based networks have been developed [see, e.g., Francis et al. (2018), Francis and Steel (2015), Huber and Scholz (2020), Pons et al. (2019), Zhang (2016)]. In addition, efficient algorithms have been presented for deciding whether or not a phylogenetic network is tree-based [see, e.g., Francis and Steel (2015) and Jetten and van Iersel (2018)]. There are also several results concerning the relationship between tree-based networks and other special classes of phylogenetic networks, as well as structural results on spaces of tree-based networks [see, e.g., Fischer and Francis (2020), Steel (2016), Corollary 10.18]. For a brief review of tree-based networks, see (Steel 2016, Section 10.4.2)

In this paper, we introduce a new class of networks called *forest-based networks*. Instead of adding arcs between edges in a phylogenetic tree, these networks are formed by adding arcs between *different* trees within a *phylogenetic forest*, i.e., a collection of leaf-disjoint phylogenetic trees (see, e.g., Fig. 1ii). Note that these networks generalize the notion of a phylogenetic network by permitting a network to have multiple roots. Forest-based networks are of interest in evolutionary studies as they can be used to model *introgression* Scholz et al. (2019), the evolutionary process in which foreign genetic material (e.g., a gene or collection of genes) is introduced into a genome Hallet (2005). Introgression is known to be common in plants and also occurs in animals. For example, in Heliconius butterflies, butterflies in one lineage can incorporate genes from butterflies in other lineages giving rise to new wing patterns Wallbank et al. (2016). To model introgression using forest-based networks, the evolutionary history of a collection of subfamilies of species (corresponding to lineages or clades) is represented by a phylogenetic forest; introgression events then correspond to the arcs that are added between different trees in the forest. The application of forest-based networks to analyzing introgression is described Scholz et al. (2019), using the special class of such networks called *overlaid species forests*.

Another potential application of forest-based networks is to the modeling of lateral gene transfer in bacteria, a process that is closely related to introgression (Hallet 2005, p.230). Lateral transfer between bacteria is commonly detected by reconciling gene trees with species trees [see, e.g., Tofigh and Hallett (2010)] which results in tree-based networks like the one in depicted in Fig. 1i. Now, suppose that each component of a phylogenetic forest is a phylogenetic tree representing the evolutionary history of a collection of bacteria living within a certain environment. Then, the forest-based network in Fig. 1ii represents how bacteria in different environments have swapped genes between one another in the past. For example, for human microbiomes, these environments could be the human mouth or gut, and the arcs between the trees in the forest would represent so-called inter-niche gene transfers Jeong et al. (2019). Forest-based networks could provide a useful additional method to standard tree reconciliation for understanding inter-environment transfers, since a phylogenetic forest can be thought of as a way to incorporate environmental constraints when trying to find optimal reconciliations. For more details, see Section 2.2 in Huber et al. (2022), where the relationship between overlaid species forests and species-gene tree reconciliation is discussed.

Although at first sight, the concept of a forest-based network appears to be a relative simple modification of the definition of a tree-based network, in this paper we shall see that its study requires the development of some interesting new theory. We now summarize the contents of the rest of this paper. In Sect. 2, we introduce basic terminology and notation. In Sect. 3, we then present the formal definition of forest-based networks and investigate some of their basic properties, for example, showing that every forest-based network has a special type of base forest (Theorem 1). In Sect. 4, we consider the relationship between forest-based *phylogenetic* networks (i.e., networks with a single root), and other well-known classes of phylogenetic networks, including tree-based networks and so-called tree-child networks Cardona et al. (2008). In Sects. 5 and 6, we consider *arboreal networks* a special class of forest-based networks whose underlying, undirected graph is a tree. In particular, in Theorem 3 we characterize arboreal networks that are forest-based, and in Theorem 6 we show that two arboreal networks induce the same set of clusters if and only if they are both forest-based.

In Sect. 7, we consider the problem of characterizing forest-based networks. More specifically, in Theorem 7 we characterize proper forest-based networks, that is, forest-based networks with $m\ge 2$ roots which are based on a phylogenetic forest which has *m* components. We also show that there is a simple characterization for forest-based networks in case $m=2$ which can be given in terms of the existence of a 2-coloring of a certain graph that can be associated to any network (Theorem 8). This is somewhat similar to the characterization of binary tree-based networks given in Jetten and van Iersel (2018). In Sect. 8, we then turn our attention to the concept of universal forest-based networks, that is networks that contain all possible phylogenetic forests as a base forest. These are a natural generalization of universal tree-based networks, which contain every possible phylogenetic tree as a base tree. Although universal tree-based networks always exist Hayamizu (2016), Zhang (2016), in Sect. 8 we show there are no universal forest-based networks with four or more leaves (Theorem 9). In Sect. 9, we conclude by presenting some potential directions for future work.

## Preliminaries

Throughout this paper, we assume that *X* is a non-empty, finite set, which can be thought of as a collection of species.

We shall use standard terminology from graph theory [see, e.g., Steel (2016), Section 1.2]. Unless stated otherwise, we assume that all graphs are directed and that they have no parallel arcs or loops. Suppose *G* is a graph. We denote the vertex set of *G* by *V*(*G*) and its set of arcs by *A*(*G*). Suppose $u,v\in V(G)$. We denote an arc *a* from *u* to *v* by $a=(u,v)$, and we refer to *u* and *v* as the *end vertices* of *a* and *v* and *u* as the *head* and *tail* of *a*, respectively. We say that *v* lies *below* *u* if there exists a directed path in *N* from *u* to *v* (so, in particular, *v* is below *v*). If, in addition, $v\not =u$ then we say that *v* lies *strictly below* *u*. We call *u* an *ancestor* of *v* if *v* is below *u*. In that case, we shall also call *v* a *descendant* of *u*. If *u* and *v* are such that (*u*, *v*) is an arc of *N*, then we call *u* a *parent* of *v* and *v* a *child* of *u*.

For $v\in V(G)$, we refer to the number of arcs with head *v* as the *indegree of v*, denoted by *indeg*(*v*), and to the number of arcs with tail *v* as the *outdegree of v*, denoted by *outdeg*(*v*). We call *v* a *leaf* of *G* if $indeg(v)=1$ and $outdeg(v)=0$, unless $V(G)=\{v\}$ in which case we also call *v* a leaf. We denote by *L*(*G*) the set of all leaves of *G*. In case *v* is not a leaf of *G* we call *v* an *internal vertex* of *G*. If $indeg(v)= 1$, then we refer to *v* as a *tree vertex* of *G*, and if $indeg(v)=0$, then we call *v* a *root* of *G*. We call every internal vertex of *G* that is neither a root nor a tree vertex a *hybrid vertex* of *G*. The set of all roots of *G* is denoted by *R*(*G*) and the set of all hybrid vertices of *G* is by *H*(*G*). We say that *G* is *semi-binary* if *G* consists of a single vertex or if every hybrid vertex of *G* has indegree two and outdegree one, and we say that *G* is *binary* if, in addition to being semi-binary, every root and every non-leaf tree vertex has outdegree two.

We say that *G* is *acyclic* if it contains no directed cycles, and we call *G* a *tree* if it has a single root, all arcs in *G* are directed away from the root, and the underlying, undirected graph of *G* is a tree (note that we regard a vertex as being a tree). We call *G* a *forest* if it has at least two connected components and all of its connected components are trees. For convenience, we will sometimes also regard a forest as being the set of trees which make up its components.

A *multiply rooted phylogenetic network* *N* (*on* *X*) or *network (on* *X*) is a semi-binary, connected, acyclic graph with leaf set *X* and at least one root, in which every root in *R*(*N*) has outdegree at least 2. In case the number $m=|R(N)|$ of roots in a network *N* is of relevance to the discussion, we sometimes also call *N* an *m*-*network (on X)*. If $N,N'$ are networks on *X*, then we say that *N* and $N'$ are *equivalent* if there exists a bijective map $\psi :V(N)\rightarrow V(N')$ that induces a graph isomorphism between *N* and $N'$ and that is the identity on *X*^1^ . If $|R(N)|=1$, then *N* is called a *phylogenetic network (on X)*. In this case, we denote the root of *N* by $\rho (N)$. If *N* is such that *H*(*N*) is empty, then we call *N* a *phylogenetic tree (on X)*. Note that in the special case where $X=\{x\}$, we regard the graph with the single vertex *x* as a phylogenetic tree on *X* with leaf and root vertex *x*. A *phylogenetic forest F (on X)* is a set consisting of at least two phylogenetic trees so that $L(T) \cap L(T') =\emptyset$ for all $T,T' \in F$, and $\bigcup _{T\in F} L(T) =X$.

We conclude this section by introducing two operations on a graph. Suppose that *G* is a graph and that $a=(u,v)$ is an arc of *G*. Then, we refer to the process of deleting *a*, adding a new vertex *w*, and adding arcs (*u*, *w*) and (*w*, *v*) as *subdividing* *a*. In this case, we also refer to *w* as a *subdivision vertex* of *a*. We call a graph $G'$ a *subdivision* of *G* if $G'$ is isomorphic to a graph that can be obtained from *G* via a finite sequence of subdivisions. Furthermore, we refer to the process that reverses subdivision (i.e., for a vertex $v\in V(G)$ with indegree and outdegree one, delete *v* and its incoming and outgoing arcs and add a new arc from the parent of *v* to the child of *v*) as *suppressing of v*. We also refer to the process that removes a root $\rho$ with outdegree 1 in a graph and the arc with tail $\rho$ as suppression.

## Forest-Based Networks

In this section, we formally define forest-based networks and present two basic results concerning their structure. Note that the concepts that we use to define a forest-based network are closely related to the ones used to define a tree-based network in Steel (2016, p.257).

**Fig. 2:**
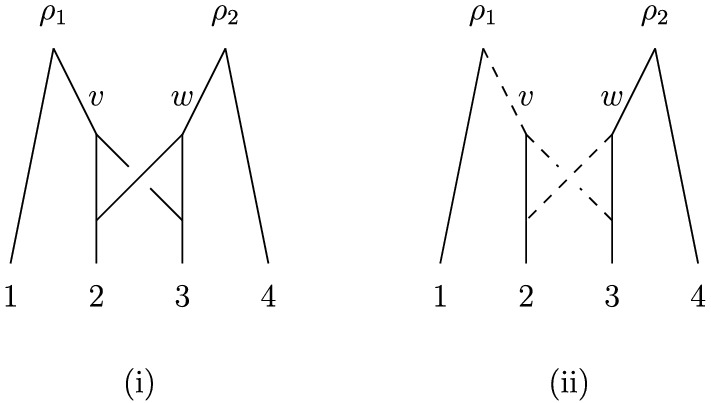
(i) A forest-based network *N* on the set $X=\{1,2,3,4\}$. For example, it is based on the phylogenetic forest *F* consisting of the isolated vertices labeled 1 and 2 and the phylogenetic tree with a single root on $\{3,4\}$. The network is also proper forest-based since it has the proper base forest consisting of the two phylogenetic trees on $\{1,2\}$ and $\{3,4\}$. (ii) An embedding of the forest *F* into *N*, where the contact arcs are indicated as dashed arcs

We define a network $N=(V,A)$ on *X* to be *forest-based* if there exists a subset $A' \subseteq A$ such that $F'=(V,A')$ is a forest with the same leaf set as *N*, and so that every arc in $A-A'$ has end vertices contained in different trees of $F'$. Note that this implies $|X| \ge 2$. We call $F'$ a *subdivision forest* for *N*, the arcs in $A-A'$ *contact arcs* and the vertices in $F'$ with indegree and outdegree both equal to one *subdivision vertices (of F)*. We call the phylogenetic forest *F* on *X* that we obtain by repeatedly suppressing all subdivision vertices and outdegree one roots in each component of $F'$ until we obtain a phylogenetic tree a *base forest* for *N*. We also say that *N* is *based on F*, and that the forest $F'$ provides an *embedding* of *F* into *N*. Note that $X=L(F)=L(F')$, and that in case a component *C* of *F* consists of a single element, then the component of $F'$ which gives rise to *C* is necessarily a path. For $m\ge 2$, we call an *m*-network *N* *proper* forest-based if it contains a *proper* base forest, that is, a base forest with *m* roots. See Fig. 2 for illustrations of these concepts.

Before proceeding, we note that not all forest-based networks are overlaid species forests, and so the concept of a forest-based network is more general than that considered in Scholz et al. (2019) (the formal definition of an overlaid species forest is quite involved and so we shall not present it here). For example, the forest-based network *N* in Fig. 2i is not an overlaid species forest. To see this, note that for every embedding of some base forest into *N*, one of the arcs with tail *v* and one of the arcs with tail *w* must be a contact arc. However, one of the conditions for a network to be an overlaid species forest is that all contact arcs must share an ancestor [cf. Huber et al. (2022), Theorem 5.3], which is not possible for any pair of contact arcs that have tail *v* and tail *w*. In the next section, we shall present some further examples and results which will elucidate the relationship between forest-based networks and various other classes of networks.

We now show that a forest-based network can be thought of as a phylogenetic forest with some arcs added in between different components of the forest (this is analogous to (Steel 2016, Proposition 10.16) for tree-based networks).

### Lemma 1

Suppose $N=(V,A)$ is a network on *X*, $|X|\ge 2$. Then, *N* is forest-based if and only if there is a set $I \subseteq A$ such that $F'=(V,A-I)$ is a forest, every arc in *I* has its end vertices in different trees of $F'$, and for every non-leaf vertex *v* of *N*, there exists an arc with tail *v* that is not contained in *I*. In particular, if *N* is *binary*, then *N* is forest-based if and only if there is a set $I \subseteq A$ such that $F'=(V,A-I)$ is a forest, every arc in *I* has its end vertices in different trees of $F'$, and for every pair of distinct arcs in *I* with a vertex *v* in common, *v* is a root of $F'$ that is not a component of $F'$.

### Proof

Suppose that *N* is forest-based. Let $I = A- A'$ for $F' = (V, A')$ some subdivision forest for *N*. Then, $F'$ is clearly a forest. Suppose *v* is a non-leaf vertex of *N*. Since $F'$ is a subdivision forest for *N*, we have that $L(F')=L(N)$. Hence, *v* is not a leaf of $F'$. Thus, there is an arc with tail *v* that belongs to $A'$.

Conversely, suppose that *I* is as in the statement of the lemma so that $F' = (V,A-I)$ is a forest. Then, clearly $L(N) \subseteq L(F' )$. Now, suppose that *v* is a non-leaf vertex of *N*. By assumption, there exists an arc with tail *v* that is not in *I*. In particular, *v* is not a leaf of $F'$, and so $L(F')=L(N)$. $\square$

Note that, as we have seen in Fig. 2, a forest-based network might have more than one base forest and different base forests for the network do not necessarily need to have the same number of components. We conclude this section by showing that every forest-based network must have a special type of base forest with |*X*| components. For *X* with $|X| \ge 2$, we define the *trivial (phylogenetic) forest* on *X* to be the phylogenetic forest in which every component is a vertex (i.e., an element of *X*).

### Theorem 1

Suppose that *N* is a network on *X*. Then, the following are equivalent

(i) *N* is forest-based.
(ii) *N* is based on the trivial forest.
(iii) The trivial forest is embedded in *N* as a union of paths (some possibly of length 0), and there is no arc in *N* joining two non-consecutive vertices of the same path.

### Proof

Clearly, (ii) implies (i), and (ii) and (iii) are equivalent.

To show that (i) implies (iii), suppose (i) holds and that *N* is forest-based with base forest *F*. Let $F'$ be an embedding of *F* such that $F'$ is not a union of paths. Then, there exists a component $C'$ of $F'$ that is not a path. Hence, $C'$ contains a vertex *v* that has outdegree greater than one, and no ancestor of *v* in $C'$ has outdegree greater than one. By removing from $C'$ all but one arc with tail *v*, we obtain an embedding $F''$ of a new base forest, such that the number of vertices of outdegree two or more in $F''$ is strictly lower than the number of vertices of outdegree two or more in $F'$. We can then repeat this process until we obtain an embedding of a forest $F_0$ such that all vertices in $F_0$ have outdegree at most one. So $F_0$ is a union of paths such that there is no arc in *N* joining two non-consecutive vertices of the same path, and therefore an embedding of the trivial forest in *N*. So (iii) holds. $\square$

### Corollary 1

Suppose that *N* is an *m*-network on *X*. If *N* is forest-based, then $|X| \ge m$. Moreover, if $|X|>m$, then *N* must contain a base forest that is not proper, and if $|X|=m$, then *N* must be proper.

### Proof

Suppose that *N* is forest-based. Then, any base forest for *N* contains at least *m* phylogenetic trees (since each root of *N* must belong to a different tree), and each of these trees has at least one leaf. So if *F* is a base forest for *N*, then $|X| \ge |F| \ge m$. The last statement now follows immediately by Theorem 1. $\square$

## Relationship of Forest-Based Networks with Other Classes of Networks

We now present some results and examples to elucidate the relationship between forest-based networks and some well-known classes of phylogenetic networks. Throughout this section, we shall focus on binary networks, as binary phylogenetic networks are commonly studied in the literature and, for some classes of phylogenetic networks, the notion of not necessarily binary can be interpreted in different ways (for example, see the remark at the end of this section concerning tree-based networks).

We begin by noting that there are networks that are neither tree-based nor forest-based [see, e.g., Steel (2016), Figure 10.10c]. Thus, it is of interest to better understand the relationship between binary forest-based networks, tree-based networks and other classes of networks. More specifically, we shall consider so-called tree-child, tree-sibling and reticulate-visible networks (see below for definitions) since, in case these have a single root, they are well-understood classes that have interesting interrelationships with tree-based networks (Steel 2016, Figure 10.12).

We begin by showing that binary forest-based *phylogenetic* networks are always tree-based.

### Proposition 1

Suppose that *N* is a binary phylogenetic network on *X*, $|X|\ge 2$. If *N* is forest-based, then it is tree-based.

### Proof

Assume that *N* is forest-based with base forest *F*, and consider the embedding $F'$ of *F* into *N*. For all trees $T'$ of $F'$ whose root $\rho _{T'}$ is distinct from the root of *N*, we can add to $F'$ the incoming arc of $\rho _{T'}$ in *N* (choosing one such arc if $\rho _{T'}$ is a hybrid vertex of *N*). Clearly, the embedding $F_0$ obtained this way is the embedding of a base tree for *N*. In particular, this means that *N* is tree-based. $\square$

We now consider tree-child networks. Generalizing the definition for tree-child phylogenetic networks Cardona et al. (2008), for a network *N* on *X*, we define *N* to be *tree-child* if all internal vertices of *N* have a child that is a tree vertex.

Note that any binary tree-child phylogenetic network is tree-based [see, e.g., Steel (2016), Corollary 10.18]. We now show that a similar result holds for binary forest-based networks.

### Theorem 2

If *N* is a binary tree-child network on *X*, $|X|\ge 2$, then it is forest-based.

### Proof

Let $I \subseteq A$ be the set of arcs (*u*, *v*) in *N* such that *v* is a hybrid vertex of *N*. If $I=\emptyset$ then *N* is a phylogenetic tree. Since a phylogenetic tree on *X* is based on the trivial phylogenetic forest, the theorem follows. So assume that $I\not =\emptyset$. Clearly, the graph $F'=(V,A-I)$ is a forest. We remark first that a leaf of $F'$ is either a leaf of *N*, or a vertex of *N* whose children are all hybrid vertices. The network *N* being tree-child, it contains no vertices of the latter type, so $L(F')=L(N)$. Moreover, all arcs (*u*, *v*) of *I* are such that *v* is a root of $F'$, so in particular *u* and *v* belong to distinct trees of $F'$. This means that $F'$ is a subdivision forest for *N*, so *N* is forest-based. $\square$

**Fig. 3:**
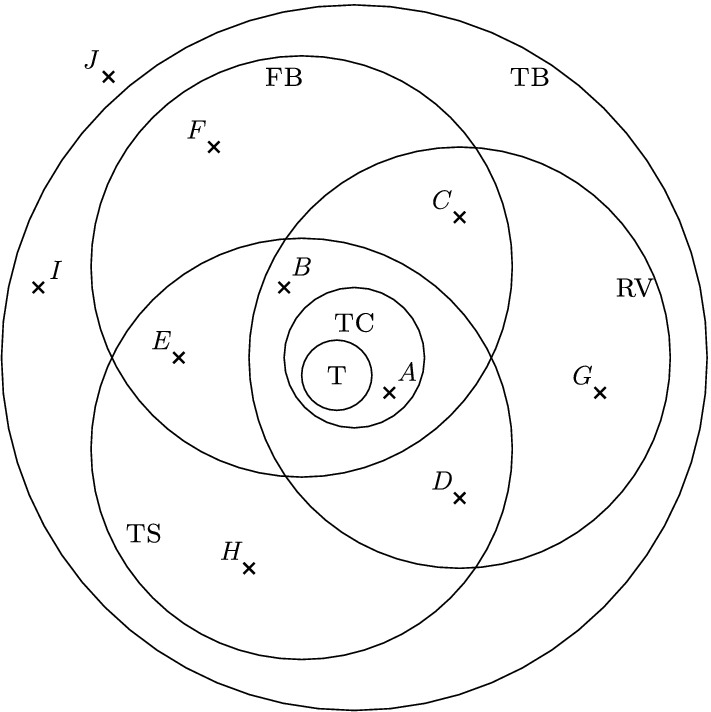
A Venn-diagram for different classes of binary phylogenetic networks; $T=\text{ tree }$, $TC=\text{ tree-child }$, $FB=\text{ forest-based }$, $TB=\text{ tree-based }$, $TS=\text{ tree-sibling }$, and $RV=\text{ reticulate-visible }$. See Fig. 4 for the indicated networks *A*–*J*

**Fig. 4:**
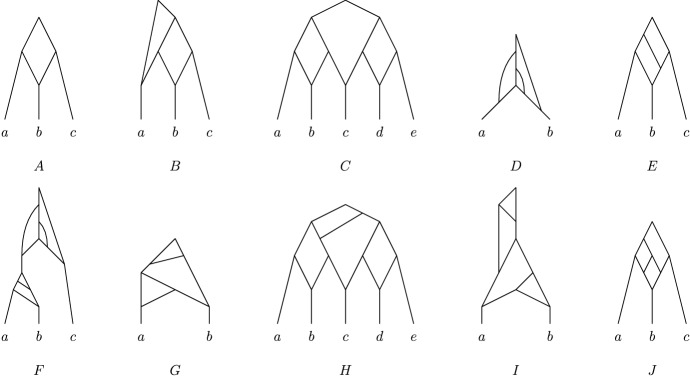
The ten phylogenetic networks used in Fig. 3

In particular, it follows from Theorem 2 that all binary tree-child *phylogenetic* networks (including phylogenetic trees!) are forest-based.

We now consider two further classes of networks. A network is (1) *tree-sibling* if for every $v \in H(N)$ there is a $v' \in V(N)$ so that $v'$ is a tree vertex and $v'$ shares a parent with *v*, and (2) *reticulation-visible* if for every $v \in H(N)$, there is a leaf $x \in X$ such that all directed paths from a root of *N* to *x* contain *v*. These definitions generalize the ones that were originally given for phylogenetic networks [see Nakhleh (2004) and Huson and Kloepper (2007), respectively]. Note that it follows immediately from the definitions that tree-child networks are tree-sibling and reticulation-visible.

In Figs. 3 and 4, we present a diagram and some examples which illustrate the interrelationship between forest-based phylogenetic networks and the other classes of phylogenetic networks that we have considered [see also Steel (2016), Figure 10.12]. Note that the network *F* in Fig. 4 provides an example which shows that we do not necessarily obtain a forest-based network by removing the root from a tree-based phylogenetic network.

We remark in passing that in Jetten and van Iersel (2018) the authors generalized the concept of a binary tree-based phylogenetic network to phylogenetic networks that are not necessarily binary in two different ways. Both versions start by adding an incoming arc to the root of a phylogenetic tree *T* and also subdivision vertices to existing arcs in *T* or in the newly added incoming arc of the root. In one version called “strictly tree-based,” arcs are added between subdivision vertices so that at most one new arc is added to a subdivision vertex. In another version called “tree-based,” arcs are added that join subdivision vertices or that start at a tree vertex and end in a subdivision vertex. In either case, subdivision vertices that have not been used are then suppressed and the root with outdegree one and its outgoing arc suppressed. As is easy to see, a 1-rooted network can be forest-based but need not be strictly tree-based. So Proposition 1 does not hold for this generalization of tree-based. Theorem 2 does, however, also hold for not necessarily binary 1-rooted networks.

## Arboreal Networks

An *arboreal* network is a network whose underlying (undirected) graph is a tree. These networks are of interest as, even though an arboreal network with more than one root has an underlying tree structure, it must still contain some reticulation vertices (since, as can be easily seen, if *N* is arboreal, then $|H(N)|=|R(N)|-1$).

**Fig. 5:**
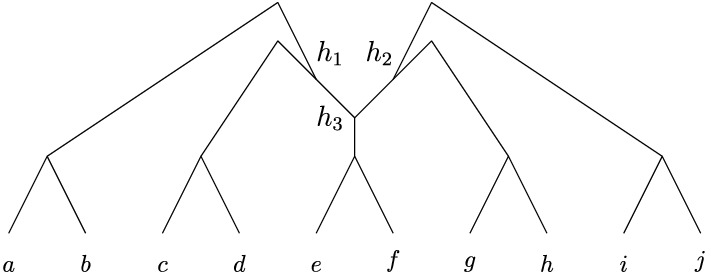
An arboreal network *N* that is not forest-based. To see this, note that one of $(h_1,h_3)$ or $(h_2,h_3)$ must be a contact arc, in which case either $h_1$ or $h_2$ becomes a leaf in the corresponding forest

In this and the next section, we shall consider properties of forest-based arboreal networks. Note that arboreal networks are not necessarily forest-based (see e. g. Fig. 5). In this section, we shall prove the following characterization for when an arboreal network is forest-based.

### Theorem 3

Suppose that *N* is an arboreal network with two or more leaves. Then, *N* is forest-based if and only if for all hybrid vertices *h* of *N*, there is a sequence of distinct vertices $v_1=h, \ldots , v_k$, $k\ge 1$, such that any two consecutive vertices in the sequence share a child that is a hybrid vertex, and $v_k$ has a child that is not a hybrid vertex. Moreover, in case this holds, then *N* is proper forest-based if and only if $|R(N)| \ge 2$.

### Proof

First note that if *N* has a single root, then $|H(N)|=|R(N)|-1=0$, and so *N* is a phylogenetic tree. Thus, it is forest-based by Theorem 2. Moreover, in this case *N* is clearly not proper forest-based. So, we shall assume from now on that $|R(N)| \ge 2$.

Suppose first that *N* is forest-based, with subdivision forest $F'$. Let *h* be a hybrid vertex of *N*. We shall associate a sequence of vertices $\sigma (h)$ to *h* and show that this sequence satisfies the properties stated in the theorem. If the child of *h* is not a hybrid vertex, then $\sigma (h)$ is the sequence that contains *h* as its sole vertex. Clearly, $\sigma (h)$ satisfies the properties stated in the theorem. So assume that the child of *h* is a hybrid vertex. Let $i\ge 1$ be such that for all $1\le j\le i$ we have that the child of $v_j$ is a hybrid vertex. Then, we define $v_{i+1}$ from $v_i$ as follows. First, we pick a child $w_i$ of $v_i$ such that $(v_i,w_i)$ is an arc of $F'$. Note that such a child must exist since $F'$ is a subdivision forest for *N*. Then, we choose $v_{i+1}$ to be a parent of $w_i$ that is not $v_i$. Note that the choice of $w_i$ implies that the arc $(v_{i+1},w_i)$ is not an arc of $F'$. In particular, we cannot have $v_{i+1}=v_{i-1}$ by the choice of *i*. Since *N* is arboreal, it follows that a given vertex of *N* cannot appear twice in $\sigma (h)$. As the number of vertices in *N* is finite, it follows that $\sigma (h)$ must end in a vertex $v_k$ that has a tree vertex as a child.

Conversely, suppose that for all hybrid vertices $h\in H(N)$, there exists a sequence $\sigma (h)$ of vertices that satisfies the stated properties. We next construct a set *I* of arcs of $N=(V,A)$ such that each arc of *I* has a hybrid vertex of *N* as head, and the graph $F'=(V,A-I)$ satisfies $L(F')=L(N)$. To do this, we start by constructing a graph $\mu (N)$ as follows: The vertices of $\mu (N)$ are all vertices of *N* with at least one child that is a hybrid vertex, and two vertices of $\mu (N)$ are joined by an edge if they share a child. We first remark that since *N* is arboreal, $\mu (N)$ does not contain cycles. We also remark that there is a trivial bijection $\chi$ between the edge set of $\mu (N)$ and *H*(*N*).

We next orient the edges of $\mu (N)$ to obtain a directed graph $\mu ^+(N)$ that has the same vertex set as $\mu (N)$. For this it suffices to consider a connected component *G* of $\mu (N)$. To this end, note that *G* is an unrooted tree and that a vertex of *G* with overall degree one is either a hybrid vertex of *N*, or a vertex of *N* with at least one child in *N* that is a tree vertex. We start by successively considering the vertices of *G* corresponding to hybrid vertices of *N* under $\chi$. Let *h* be such a vertex of *N*. Then, by assumption, sequence $\sigma (h) = (v_1=h, v_2,\ldots , v_k)$, $k\ge 1$, satisfies the properties stated in the theorem. For $i \in \{1, \ldots , k-1\}$ and all $j\in \{1,\ldots , i-1\}$, assume that the edges $\{v_j,v_{j+1}\}$ have already been oriented, and that the edge $e=\{v_i,v_{i+1}\}$ has not yet been assigned an orientation. Then, we direct *e* from $v_i$ to $v_{i+1}$.

Once all vertices on $\sigma (h)$ have been processed, we orient all edges of *G* whose tail is a vertex *v* in $\sigma (h)$ and which have not already been processed away from *v*. Repeating this process for all vertices that are heads in the resulting graph and so on results in an oriented graph $\mu ^+(N)$. Note that this includes the case where *G* does not contain any vertex corresponding to a hybrid vertex of *N*. By construction, our assumptions on $\sigma (h)$ imply that a vertex of *G* of outdegree 0 has a child in *N* that is a tree vertex, as desired. Furthermore, $\chi$ induces a natural bijection $\chi ^+$ between the arc set of $\mu ^+(N)$ and *H*(*N*).

Armed with $\mu ^+(N)$, we construct a set *I* of arcs of *N* as follows. First, we initialize *I* with the empty set. Next, for each hybrid vertex *h* of *N*, we add the arc $(v,h)\in A$ to *I*, where *v* is the head of the arc in $\mu ^+(N)$ corresponding to *h* under $\chi ^+$.

Clearly, the graph $F'=(V,A-I)$ is a forest, since it contains exactly one incoming arc for each hybrid vertex of *N*. To see that $L(F')=L(N)$, it suffices to remark that each non-leaf vertex *v* of *N* has an outgoing arc in $F'$. If *v* has at least one child that is not a hybrid vertex, then the set equality holds. Otherwise, *v* is a vertex of $\mu (N)$ whose indegree in $\mu ^+(N)$ is at least one. By definition of *I*, for *h* a hybrid vertex corresponding to an outgoing arc of *v* in $\mu ^+(N)$ under $\chi ^+$, the arc (*v*, *h*) is an arc of $F'$.

To conclude that $F'$ is a subdivision forest for *N*, it suffices to remark that since *N* is arboreal, there exists no arc in *I* whose both end vertices are in the same tree of $F'$. So *N* is forest-based. Moreover, we have that $|R(N)|=|R(F')|$. Thus, *N* is proper forest-based. $\square$

## Cluster Systems from Arboreal, Forest-Based Networks

In phylogenetics, it is common to work with rooted phylogenetic trees in terms of clusters that they induce as these can be sometimes easier to handle (e.g., for consensus methods or for computing distances between phylogenetic trees—cf., e.g., [(Steel (2016), Section 2.2.2]. We can also associate clusters to networks as follows. Suppose *N* is a network on *X* and $u\in V(N)$. We call the set $C(u)=C_N(u)$ of leaves of *N* below *u* the *cluster* induced by *u*. If $|C_N(u)|=1$ then we call $C_N(u)$ a *trivial cluster (on X)*. We refer to the set $\mathscr {C}(N)$ of all clusters induced by the vertices in *V*(*N*) as the *cluster system* induced by *N* and, more generally, we also refer to any collection of non-empty subsets or clusters in *X* by the same name.

**Fig. 6:**
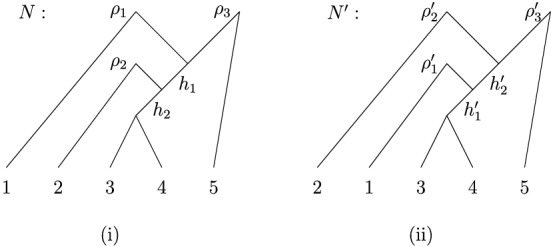
Two arboreal, 3-rooted networks *N* and $N'$. Note that ${\mathscr {C}}(N)={\mathscr {C}}(N')$, but that *N* and $N'$ are not equivalent. The bad arcs are the arcs $(h_1,h_2)$ and $(h_2',h_1')$

Interestingly, in contrast to phylogenetic trees, there are non-equivalent arboreal networks that have the same cluster systems (see e. g. Fig. 6). Even so, in this section we shall show that if *N* and $N'$ are distinct arboreal networks with ${\mathscr {C}}(N)={\mathscr {C}}(N')$, then *N* is forest-based if and only if $N'$ is forest-based (Theorem 6). To do this, we will first prove two equivalence results for arboreal networks (Theorems 4 and  5) that are analogous to the well-known equivalence theorem between phylogenetic trees and hierarchies. This latter result states that, given a cluster system ${\mathscr {C}}$, there is a phylogenetic tree *T* on *X* such that $\mathscr {C}(T)={\mathscr {C}}$ if and only if ${\mathscr {C}}$ is a *hierarchy (on X)* (that is, ${\mathscr {C}}$ contains all trivial clusters and *X* and, for all $C, C' \in {\mathscr {C}}$, $C \cap C' \in \{C,C',\emptyset \}$) and that, if such a phylogenetic tree *T* exists, then up to equivalence, *T* is uniquely determined by ${\mathscr {C}}(T)$ [see e. g.  Steel (2016), Proposition 2.1].

To state our first result, we require further definitions. We say that an arboreal network *N* is *uniquely determined* by ${\mathscr {C}}(N)$ if any arboreal network $N'$ for which $\mathscr {C}(N)={\mathscr {C}}(N')$ holds is equivalent to *N*. Furthermore, for $v\in R(N)$, we denote by *T*(*v*) the subtree of *N* spanned by all leaves below *v*, and we denote by $T_v$ the phylogenetic tree obtained from *T*(*v*) by suppressing all vertices *u* with $indeg({u})= 1= outdeg(u)$. Given a cluster system ${\mathscr {C}}$ on *X*, we denote by ${\mathscr {I}}({\mathscr {C}})$ the graph whose vertex set is ${\mathscr {C}}$ and whose edge set is the set of pairs $\{C,C'\}\in {{\mathscr {C}}\atopwithdelims ()2}$ such that $C \cap C' \ne \emptyset$, and by ${\mathscr {C}}_M\subseteq {\mathscr {C}}$ the collection of set-inclusion maximal elements of ${\mathscr {C}}$.

### Theorem 4

Let ${\mathscr {C}}$ be a cluster system on *X*. Then, there exists an arboreal $| {\mathscr {C}}_M|$-rooted network *N* such that $\mathscr {C}(N)={\mathscr {C}}$ if and only if:

1. For all $C \in {\mathscr {C}}_M$, the set $\{C' \in {\mathscr {C}}\,:\, C' \subseteq C\}$ is a hierarchy that contains all trivial clusters on *C*.
2. The graph ${\mathscr {I}}({\mathscr {C}}_M)$ is connected.
3. For any two $C_1,C_2 \in {\mathscr {C}}_M$, we have $C_1 \cap C_2 \in {\mathscr {C}} \cup \{\emptyset \}$.

To establish this result, we will use the following lemma:

### Lemma 2

Let *N* be an arboreal network on *X*. Then, the set inclusion maximal elements of ${\mathscr {C}}(N)$ are precisely the clusters *C*(*r*) with $r \in R(N)$.

### Proof

If $|X|=1$ then the lemma trivially holds. So assume that $|X|\ge 2$. Clearly, all set-inclusion maximal elements *C* of $\mathscr {C}(N)$ are such that $C=C(r)$, for some $r \in R(N)$. Assume for contradiction that there exists a root $r \in R(N)$ such that *C*(*r*) is not set-inclusion maximal in ${\mathscr {C}}(N)$. Then, there must exists $v \in V(N)$ such that $C(r) \subsetneq C(v)$. Hence, for all $x\in C(r)$, there exists a directed path from *v* to *x*. Since *v* cannot be an ancestor of *r* (as *r* is a root of *N*), it follows that *v* and *r* are vertices in a cycle in the underlying undirected graph of *N* which contradicts the assumption that *N* is arboreal. $\square$

### Proof of Theorem 4

Since the theorem clearly holds if $|X|=1$, we may assume that $|X|\ge 2$. Put $m=| {\mathscr {C}}_M|$. Assume first that there exists an arboreal *m*-network *N* such that ${\mathscr {C}}(N)={\mathscr {C}}$. By Lemma 2, we have ${\mathscr {C}}_M=\{C(r)\,|\, r\in R(N)\}$. Let $C \in {\mathscr {C}}_M$ and let *r* be the root of *N* such that $C=C(r)$.

Then, since *N* is arboreal, $T_r$ is a phylogenetic tree on some subset $X_r$ of *X*. Hence, ${\mathscr {C}}(T_r)$ is a hierarchy on $X_r$. Since ${\mathscr {C}}(T_r) = \{C' \in {\mathscr {C}}\,|\, C' \subseteq C\}$, it follows that Property (P1) must hold.

To see that Property (P2) holds, Assume first that $|\mathscr {C}_M|=1$. Since a graph consisting of a single vertex is connected, the theorem holds. So assume that $|{\mathscr {C}}_M|\ge 2$. Let *r* and $r'$ be two roots of *N*. Since *N* is connected, there exists an undirected path in *N* between *r* and $r'$. Let $h_1, \ldots , h_k$, $k \ge 1$, be the hybrid vertices of *N* successively crossed by that path. For all $1 \le i \le k$, all sets in ${\mathscr {C}}_M$ corresponding to roots that are ancestors of $h_i$ form a clique in ${\mathscr {I}}({\mathscr {C}}_M)$, since they all contain the cluster $C(h_i)$. Since *r* is an ancestor of $h_1$ and $r'$ an ancestor of $h_k$, it follows that there exists a path in ${\mathscr {I}}(\mathscr {C}_M)$ joining *C*(*r*) and $C(r')$. Hence, ${\mathscr {I}}({\mathscr {C}}_M)$ is connected and Property (P2) holds.

To see that Property (P3) holds, let $C_1$ and $C_2$ be two elements of ${\mathscr {C}}_M$, and let $r_1$ and $r_2$ be the roots of *N*, so that $C_1=C(r_1)$ and $C_2=C(r_2)$. Consider the set $H_{1,2}$ of all hybrid vertices that are below both $r_1$ and $r_2$. If $H_{1,2}=\emptyset$, then $C_1 \cap C_2= \emptyset$. If $|H_{1,2}| \ge 1$, then since *N* is arboreal, there exists a directed path in *N* containing all vertices in $H_{1,2}$. In particular, there is a vertex $h \in H_{1,2}$ that is an ancestor of all vertices of $H_{1,2}$ in *N*. This vertex *h* satisfies $C(h)=C_1 \cap C_2$, and so $C_1 \cap C_2 \in {\mathscr {C}}$. Thus, Property (P3) holds.

Conversely, assume that ${\mathscr {C}}$ is a cluster system on *X* that satisfies Properties (P1)–(P3). Then, for all $C \in {\mathscr {C}}_M$, Property (P1) implies that the set ${\mathscr {C}}_C=\{C' \in \mathscr {C}\,|\, C' \subseteq C\}$ is a hierarchy on *C* that contains all trivial clusters on *C*. Hence, by the remark above, there exists a unique (up to equivalence) phylogenetic tree *T*(*C*) on *C* such that ${\mathscr {C}}(T(C))={\mathscr {C}}_C$. Put $F=\{T(C)\,|\, C \in \mathscr {C}_M\}$, and note that *F* need not be a phylogenetic forest on *X* since the leaf sets of the trees in *F* might not be pairwise disjoint.

We next use the trees in *F* to recursively construct an arboreal *m*-rooted network *N* such that ${\mathscr {C}}(N)={\mathscr {C}}$. Put $F=\{T_1,\ldots , T_m\}$. First, let $N_1$ be some tree in *F* which, without loss of generality, we may assume to be $T_1$. Clearly, *T* is an arboreal 1-network. Let $1 \le i < m$ and assume that, for all $1\le j\le i$, we have already constructed an arboreal *j*-network $N_j$ by processing (subject to potentially having to relabel the trees in *F*) the tree $T_j\in F$. We now construct an arboreal $(i+1)$-network $N_{i+1}$ from $N_i$ as follows.

First, we choose a tree $T\in F-\{T_1, \ldots , T_i\}$ such that ${\mathscr {C}} (T) \cap {\mathscr {C}}(N_i) \ne \emptyset$,

Note that it is always possible to find such a tree *T* due to the connectivity of ${\mathscr {I}}({\mathscr {C}}_M)$ that is guaranteed by Property (P2). Also note that we may assume without loss of generality that $T=T_{i+1}$. Because of Property (P3), there exists exactly one tree vertex $u_i$ in $N_i$ and one vertex $v_{i+1}$ in *T* such that $C_{N_i}(u_i)=C_{T}(v_{i+1})$.

If $u_i$ were a root of $N_i$ then since, by Lemma 2, $C_{N_i}(u_i)$ is a maximal cluster for $N_i$ it follows that $C_T(v_{i+1})$ is also a maximal cluster of $N_i$. The definition of *F* implies that $T(C_{N_i}(u_i))=T(C_T(v_{i+1}))=T$ which is impossible as *T* has not been processed yet. So $u_i$ cannot be a root of $N_i$. We then define $N_{i+1}$ as the $(i+1)$-rooted directed graph obtained from $N_i$ by subdividing the incoming arc of $u_i$ in $N_i$ by a vertex *w*, removing all arcs and vertices below $v_{i+1}$ in *T*, and identifying $v_{i+1}$ with *w*.

By construction, $N_{i+1}$ is clearly a $(i+1)$-network satisfying ${\mathscr {C}}(N_{i+1})=\bigcup _{1 \le j \le i+1} {\mathscr {C}}(T_j)$. Furthermore, since $N_i$ is arboreal $N_{i+1}$ must also be arboreal. In particular, this implies that $N=N_m$ is a *m*-rooted network satisfying ${\mathscr {C}}(N_m)={\mathscr {C}}$. This concludes the proof. We now turn to the question of uniqueness. Note that the construction of a network *N* from a cluster system ${\mathscr {C}}$ on *X* satisfying Properties (P1)–(P3) as described in the proof of Theorem 4 requires choices to be made (e.g., the order in which the trees in the forest are processed in case there is a tie). As a consequence, the resulting network *N* satisfying $\mathscr {C}(N)={\mathscr {C}}$ need not be unique. This issue is illustrated in Fig. 6. However, defining an arc in a network to be *bad* if both of its end vertices are contained in *H*(*N*), we have the following result. To help establish it, we denote by *Comp*(*M*) the multiply rooted graph obtained from an arboreal network *M* by collapsing all bad arcs of *M*.

$$\square $$

### Proposition 2

Let *N* be an arboreal network on *X*. Then, *N* is uniquely determined by ${\mathscr {C}}(N)$ if and only if *N* contains no bad arcs.

### Proof

Clearly, the results hold if $|X|=1$. So assume $|X|\ge 2$. Suppose first that *N* is uniquely determined by ${\mathscr {C}}(N)$. Assume for contradiction that *N* contains a bad arc (*u*, *v*) with $u,v \in V(N)$. Let $p_u$ be a parent of *u*, and let $p_v$ be the parent of *v* distinct from *u*. Because *N* is arboreal, there is no directed path either from $p_u$ to $p_v$ or from $p_v$ to $p_u$. Consider the network $N'$ on *X* obtained from *N* by replacing the arcs $(p_u,u)$ and $(p_v,v)$ with the arcs $(p_u,v)$ and $(p_v,u)$. Since $C_N(u)=C_N(v)$ it follows that ${\mathscr {C}}(N')=\mathscr {C}(N)$. However, *N* and $N'$ are not equivalent. This is a contradiction since, by assumption, *N* is uniquely determined by ${\mathscr {C}}(N)$.

Conversely, suppose that *N* does not contain a bad arc. Assume for contradiction that there exists an arboreal network $N'$ on *X* such that ${\mathscr {C}}(N')={\mathscr {C}}(N)$ but *N* and $N'$ are not equivalent. Since a hybrid vertex in a network on *X* induces the same cluster on *X* as its child, it follows that there must exist a bijection $\chi$ between the set *T*(*N*) of root and tree vertices of *N* and the set $T(N')$ of root and tree vertices of $N'$ such that $C_N(v)=C_{N'}(\chi (v))$, for all $v\in T(N)$. Note that *N* must be equivalent to *Comp*(*N*) because *N* does not contain a bad arc. Then, since *N* and $N'$ are not equivalent, *Comp*(*N*) and $Comp(N')$ are also not equivalent. We distinguish the cases that $Comp(N')= N'$ and that $Comp(N')\ne N'$.

If $Comp(N')$ is $N'$, then there must be two tree vertices *u* and *v* in *N* and a vertex $h\in H(N')$ such that (*u*, *v*) is an arc in *N* and $\chi (u),h,\chi (v)$ is a directed path in $N'$. So there must exist some $w\in T(N')-\{\chi (u)\}$ such that *w* is a parent of *h*. Note that $u\not =\chi ^{-1}(w)$. If *u* were strictly below $\chi ^{-1}(w)$ then $C_N(u)\subsetneq C_N(\chi ^{-1}(w))$. Hence, $C_N(v)= C_{N'}(\chi (v))=C_{N'}(\chi (u))\cap C_{N'}(w)= C_N(u)\cap C_N(\chi ^{-1}(w))=C_N(u)$ and so $C_N(v)=C_N(u)$, a contradiction as (*u*, *v*) is an arc in *N* and so $C_N(v)\not = C_N(u)$. Similar arguments also imply that $\chi ^{-1}(w)$ cannot be strictly below *u*. Since $C_N(v)=C_{N'}(\chi (v))\subseteq C_{N'}(w)$ it follows that $C_N(v)=C_N(u)\cap C_N(\chi ^{-1}(w))=\emptyset$, which is again a contradiction.

If $Comp(N')$ is not $N'$, then $N'$ must contain a bad arc, say $(h_1,h_2)$, $h_1, h_2 \in H(N)$. Without loss of generality, we may assume that $h_1$ and $h_2$ are such that the child *w* of $h_2$ in $N'$ is a tree vertex. Let $r_1$ and $r_2$ be two distinct roots of $N'$ that are ancestors of $h_1$ and let $r_3$ be a root of $N'$ that is an ancestor of $h_2$ but not of $h_1$. Then, $C_{N'}(r_1) \cap C_{N'}(r_2)=C_{N'}(r_1) \cap C_{N'}(r_3)=C_{N'}(w)$. It follows that there must exist some tree vertex $w_1\in V(N)$ such that $C_N(\chi ^{-1}(r_1)) \cap C_N(\chi ^{-1}(r_2))=C_N(\chi ^{-1}(r_1)) \cap C_N(\chi ^{-1}(r_3))= C_N(w_1)$. Consequently, the parent *p* of $w_1$ and the parent of *p* are both hybrid vertices of *N*. But then *N* contains a bad arc, acontradiction. $\square$

Interestingly, the existence of bad arcs in *N* can be determined by the relations between the maximal elements of ${\mathscr {C}}(N)$.

### Lemma 3

Let *N* be an arboreal network on *X* and let $\mathscr {C}={\mathscr {C}}(N)$. Then, *N* contains a bad arc if and only if there exists $C_1, C_2, C_3 \in {\mathscr {C}}_M$ distinct such that $C_1 \cap C_2=C_1 \cap C_3= C_2 \cap C_3 \ne \emptyset$.

### Proof

Suppose first that *N* has a bad arc $(h_1,h_2)$. Let $v_1,v_2$ be the parents of $h_1$, and let $r_1,r_2$ be two roots of *N* that are ancestors of $v_1$ and $v_2$ respectively. Finally, let $r_3$ be a root that is an ancestor of the parent $v_3$ of $h_2$ distinct from $h_1$. Since *N* is arboreal, $r_1, r_2$ and $r_3$ are all distinct. By Lemma 2, we have that $C_1=C(r_1), C_2=C(r_2)$ and $C_3=C(r_3)$ all belong to ${\mathscr {C}}_M$. Moreover, using again the fact that *N* is arboreal, we have $C_1 \cap C_2=C(h_1)$ and $C_1 \cap C_3=C_2 \cap C_3=C(h_2)$. Clearly, $C(h_i)\not =\emptyset$, for all $i=1,2,3$. Moreover, since $h_2$ is the unique child of $h_1$ in *N*, we have $C(h_1)=C(h_2)$. Thus, $C_1 \cap C_2=C_1 \cap C_3= C_2 \cap C_3 \ne \emptyset$.

Conversely, suppose that there exists $C_1, C_2, C_3 \in {\mathscr {C}}_M$ distinct such that $C_1 \cap C_2=C_1 \cap C_3=C_2 \cap C_3 \ne \emptyset$. By Lemma 2, there exists three roots $r_1,r_2, r_3$ of *N* such that $C_1=C(r_1)$, $C_2=C(r_2)$ and $C_3=C(r_3)$. Since *N* is arboreal, $C_1 \cap C_2 \ne \emptyset$ implies that there exists a hybrid vertex $h_a$ of *N* whose parents are descendants of $r_1$ and $r_2$, respectively. For the same reason, there exists a hybrid vertex $h_b$ of *N* whose ancestors are descendants of $r_1$ and $r_3$, respectively, and a hybrid vertex $h_c$ of *N* whose ancestors are descendants of $r_2$ and $r_3$, respectively. Note that we have $C(h_a)=C_1 \cap C_2$, $C(h_b)=C_1 \cap C_3$ and $C(h_c)=C_2 \cap C_3$. Hence, by assumption, $C(h_a)=C(h_b)=C(h_c)$. Clearly, $h_a=h_b=h_c$ cannot hold. Hence, there exists two distinct hybrid vertices $h_1$ and $h_2$ of *N* such that $C(h_1)=C(h_2)$. Since *N* is arboreal, this implies that there exists a path in *N* from $h_1$ to $h_2$ (or from $h_2$ to $h_1$) containing only hybrid vertices. In particular, all arcs on such a path are bad arcs. So, *N* contains at least one bad arc. $\square$

Armed with these results, we obtain the following:

### Theorem 5

Let ${\mathscr {C}}$ be a cluster system on *X* that satisfies Properties (P1)–(P3) and contains all trivial clusters on *X*. Then, there exists an arboreal network *N* on *X* that satisfies ${\mathscr {C}}(N)= {\mathscr {C}}$. Moreover, if such an aboral network *N* exists then, up to equivalence, *N* is uniquely determined by ${\mathscr {C}}$ if and only there exists no $C_1, C_2, C_3 \in {\mathscr {C}}_M$ distinct such that $C_1\cap C_2=C_1 \cap C_3= C_2 \cap C_3$. Moreover, if *N* and $N'$ are two arboreal networks on *X*, then ${\mathscr {C}}(N)={\mathscr {C}}(N')$ if and only if *N* and $N'$ are equivalent after collapsing all bad arcs.

### Proof

Let *N* be an arboreal network on *X* satisfying $\mathscr {C}(N)={\mathscr {C}}$. Note that *N* exists by Theorem 4. We have that *N* is uniquely determined by ${\mathscr {C}}$ if and only if *N* contains no bad arc (Proposition 2). By Lemma 3, *N* contains no bad arc if and only if there exists no $C_1, C_2, C_3 \in {\mathscr {C}}_M$ distinct such that $C_1\cap C_2=C_1 \cap C_3= C_2 \cap C_3$. Hence, the desired equivalence follows

To see the final part of the theorem, let *N* be an arboreal network satisfying ${\mathscr {C}}(N)={\mathscr {C}}$, and let $r_1, \ldots , r_m$, $m \ge 1$ denote the roots of *N*. Note that *Comp*(*N*) is uniquely determined by $\bigcup _{i=1}^m{\mathscr {C}}(T_i)$ where, for all $1\le i\le m$, $T_i$ is the subtree of *N* rooted at $r_i$. In particular, *Comp*(*N*) does not depend on the choice of *N* among the arboreal networks $N'$ with ${\mathscr {C}}={\mathscr {C}}(N')$. $\square$

We now prove the main result of this section.

### Theorem 6

Let $N_1$ and $N_2$ be two distinct arboreal networks on *X* with ${\mathscr {C}}(N_1)={\mathscr {C}}(N_2)$. Then, $N_1$ is forest-based if and only if $N_2$ is forest-based.

### Proof

If $|X|=1$, then the theorem clearly holds. So assume that $|X|\ge 2$. Without loss of generality, it suffices to show that if $N_1=(V_1,A_1)$ is forest-based, then $N_2=(V_2,A_2)$ must be forest-based too. So assume that $N_1$ is forest-based, with subdivision forest $F_1'=(V_1,A_1')$. Set $I_1=A_1-A_1'$.

Since, by assumption, ${\mathscr {C}}(N_1)={\mathscr {C}}(N_2)$ Theorem 5 implies that $N_1$ and $N_2$ are equivalent after collapsing all bad arcs. Let $N_0$ be the graph obtained from $N_1$ in this way. Note that $N_0$ is not a network in our sense, as it need not be semi-binary. Clearly, no arc in $I_1$ has a hybrid vertex as tail, so all arcs in $I_1$ are arcs of $N_0$. Since $N_0$ can also be obtained by collapsing all bad arcs of $N_2$, this induces a trivial bijection $\chi$ between $I_1$ and some set $I_2$ of arcs of $N_2$.

It remains to show that the forest $F_2'=(V_2,A_2-I_2)$ is a subdivision forest for $N_2$. Clearly, we have that $L(N_2) \subseteq L(F_2')$ and, since $N_2$ is arboreal, no arc of $I_2$ joins two vertices from the same tree in $F_2'$. To see that $L(F_2') \subseteq L(N_2)$ holds too, assume for contradiction that there is a vertex $v_2\in L(F_2')-L(N_2)$. Then, all arcs of $N_2$ with tail $v_2$ are in $I_2$. Note that $I_2$ has been defined in such a way that no arc in $I_2$ is collapsed when transforming $N_2$ into $N_0$. Moreover, the property of having a vertex such that all outgoing arcs belong to a given set is preserved when resolving vertices of a network. Since $\chi$ is the trivial bijection between $I_1$ and $I_2$, it follows that, there must exist a vertex $v_1$ in $N_1$ such that all arcs of $N_1$ with tail $v_1$ are in $I_1$. This is a contradiction since $F_1'=(V_1,A_1')$ is a subdivision forest for $N_1$ and so $L(F_1')=L(N_1)$. Hence, $N_2$ is forest-based. $\square$

## Characterizing Proper Forest-Based Networks

**Fig. 7:**
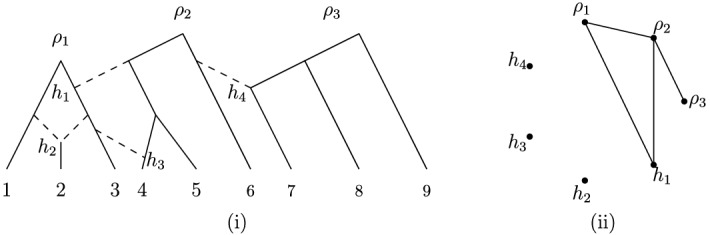
(i) A 3-rooted forest-based network *N* with ${\mathfrak {O}}(N)=\emptyset$ that is not proper forest-based. (ii) The graph $\Gamma (N)$

**Fig. 8:**
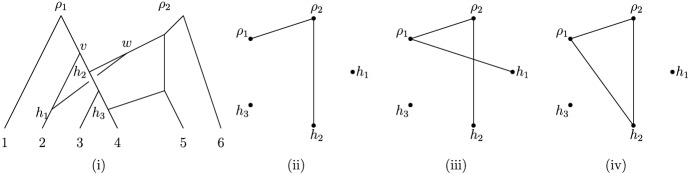
(i) A 2-rooted forest-based network *N* with ${\mathfrak {O}}(N)=\{v,w\}$. (ii) The graph $\Gamma (N)$. (iii) and (iv) Two distinct omni-extensions of $\Gamma (N)$, with a minimum number of possible edges. Since one of these extensions has no cycle of length 3, it follows by Theorem 8 that *N* is proper forest-based

In this section, we present two characterizations for proper forest-based networks (Theorems 7 and 8). Various characterizations have been given for tree-based *phylogenetic* networks [see, e.g., Francis and Steel (2015) and Steel (2016), Theorem 10.17]. Some of these are given in terms of bipartite graphs, one of which from Jetten and van Iersel (2018) we now recall. Define a vertex in a network *N* to be an *omnian (vertex)* of *N* if all of the children of *v* are contained in *H*(*N*), and let ${\mathfrak {O}}(N)$ denote the set of omnians in *N* (see e. g.  Figs. 7i and 8i). To a network *N* associate the bipartite graph $(U \cup H, E)$, where *U* contains a vertex $u_v$ for each omnian $v \in {\mathfrak {O}}(N)$, *H* contains a vertex $u_w$ for each hybrid vertex $w \in H(N)$, and *E* consists of the edges $\{u_v,u_w\}$ such that there is a some $v \in {\mathfrak {O}}(N)$ and some $w \in H(N)$ with (*v*, *w*) an arc in *N*. Then, a binary *phylogenetic* network is tree-based if and only if $(U \cup H, E)$ contains a matching *M* such that $|M|=|U|$ [Jetten and van Iersel 2018, Theorem 2.4]. Interestingly, we found that characterizing forest-based networks is more subtle although, as we shall now see, we can still characterize proper forest-based networks using omnians.

To this end, we introduce some further definitions. Suppose that *N* is a network and that $v \in V(N)$. We define the vertex $\gamma _v\in RH(N)=R(N) \cup H(N)$ to be the (unique) ancestor of *v* such that no vertex in $RH(N)-\{\gamma _v\}$ is contained in the directed path *P* from $\gamma _v$ to *v* (e.g., in Fig. 7i.

for leaf 5, $\gamma _5=\rho _2$, and for leaf 3, $\gamma _3=h_1$). Note that $\gamma _v=v$ if and only if $v \in RH(N)$. The rationale behind the definition of $\gamma _v$ is that, for any base forest *F* in a proper forest-based network, the vertices *v* and $\gamma _v$ must belong to the same tree in *F*.

We next associate an undirected graph $\Gamma (N)$ to *N* (which may also contain loops). The vertex set of $\Gamma (N)$ is the set *RH*(*N*), and (not necessarily distinct) vertices $u, v \in RH(N)$ form an edge $\{u,v\}$ in $\Gamma (N)$ if there exists a hybrid vertex $h\in H(N)$ with parents $u'$ and $v'$ such that $u=\gamma _{u'}$ and $v=\gamma _{v'}$ (see, e.g., Fig. 7ii). In addition, we call any (undirected) supergraph $\Gamma '(N)$ of $\Gamma (N)$ with the same vertex set as $\Gamma (N)$ an *omni-extension* of $\Gamma (N)$ if, for any omnian $v \in {\mathfrak {O}}(N)$, there exists a child *h* of *v* such that $\{\gamma _u, h\}$ is an edge of $\Gamma '(N)$ for *u* the second parent of *h* (see, e.g., Fig. 8iii).

Note that there exist networks *N* on *X* such that $\Gamma (N)$ has more than one omni-extension (e.g., Fig. 8), and also that if *N* does not contain any omnians, then $\Gamma (N)$ is an omni-extension of itself (this can also hold even if *N* contains omnians). We will use the following useful additional observation concerning omni-extensions to obtain our characterization of proper forest-based networks.

### Lemma 4

Let *N* be a *m*-rooted network on *X*, some $m\ge 2$. If *N* is proper forest-based with proper base forest *F*, then $\Gamma (N)$ has an omni-extension that does not contain loops, namely the graph $\Gamma _F(N)$ having the same vertex set as $\Gamma (N)$, and with edge set consisting of those $\{u,v\}$, $u,v\in RH(N)$, such that *u* and *v* belong to different trees in *F*.

### Proof

We first establish that $\Gamma (N)$ is a subgraph of $\Gamma _F(N)$. Suppose $e=\{u,v\}$ with $u,v\in RH(N)$ is an edge in $\Gamma (N)$. Then, there is a hybrid vertex in *N* with parents $u'$ and $v'$ such that $u=\gamma _{u'}$ and $\gamma _{v'}=v$. Since *N* is based on *F*, $u'$ and $v'$ must belong to two different trees in *F*. Since *F* is a proper base forest for *N*, the vertices $\gamma _{u'}$ and $\gamma _{v'}$ must belong to two different trees in *F*. Thus, $u\not = v$. By definition of $\Gamma _F(N)$, it follows that *e* is an edge of $\Gamma _F(N)$.

To show that $\Gamma _F(N)$ is an omni-extension of $\Gamma (N)$, consider an omnian *v* of *N*. As *N* is based on *F*, *v* must have at least one child *h* such that *v* and *h* belong to the same tree $T_v$ of *F*. Hence, for *u* the parent of *h* other than *v*, *u* does not belong to $T_v$. Thus, $\gamma _u$ and *h* belong to different trees in *F* because *F* is a proper base forest for *N*. Hence, $\{\gamma _u,h\}$ is an edge of $\Gamma _F(N)$. $\square$

We now present our characterization for proper forest-based networks. Recall that if *G* is a undirected graph (possibly with loops), and *Y* is a non-empty set of colors, then a map $\sigma : V(G) \rightarrow Y$ satisfying $\sigma (u) \ne \sigma (v)$ for all edges $\{u,v\}$ of *G* is a *proper vertex coloring* of *G*. Moreover, if there exists such a coloring with $|Y|=k \ge 1$, then *G* is called *k*-*colorable*; if $k=2$ then *G* is *bipartite*.

### Theorem 7

Let *N* be a *m*-rooted network on *X*, some $m\ge 2$, and let $\{s_1,\ldots , s_m\}$ be a set of *m* colors. Then, *N* is proper forest based if and only if there exists an omni-extension $\Gamma '(N)$ of $\Gamma (N)$ and a proper vertex coloring $\sigma : RH(N) \rightarrow \{s_1, \ldots , s_m\}$ of $\Gamma '(N)$ satisfying:

1. The restriction of $\sigma$ to *R*(*N*) is a bijection.
2. For all $u \in R(N)$ and all $v \in H(N)$ such that $\sigma (u)=\sigma (v)$ there must exist a directed path *P* in *N* from *u* to *v* such that $\sigma (w)=\sigma (u)$ holds for all vertices $w \in H(N)$ that lie on *P*.

### Proof

Assume first that *N* is proper forest-based with proper base forest *F*. Let $\Gamma _F(N)$ be the omni-extension of $\Gamma (N)$ given in Lemma 4. Let $\sigma _F: RH(N) \rightarrow R(N)$ be the map that assigns to every vertex $v \in RH(N)$ the unique root $\rho$ of *N* such that the directed path from $\rho$ to *v* does not contain a contact arc of *N*. Note that such a path may consist of a single vertex. Since *N* is based on *F*, it follows that $\sigma _F$ is well-defined and a proper vertex coloring of $\Gamma _F(N)$. By definition, $\sigma _F$ satisfies Properties (C1) and (C2).

Conversely, let $\Gamma '(N)$ be an omni-extension of $\Gamma (N)$, let $S=\{s_1, \ldots , s_m\}$ denote a set of *m* colors, and let $\sigma : RH(N) \rightarrow S$ be a proper vertex coloring of $\Gamma '(N)$ that satisfies Properties (C1) and (C2). For $1 \le i \le m$, let $T_i$ denote the subgraph of *N* induced on the set $V'$ of vertices *v* in *N* with $\sigma (\gamma _v)=s_i$ (that is, the graph with vertex set $V'$ and arc set $\{(u,v) \in A(G): u, v \in V'\}$).

Suppose $i\in \{1,\ldots , m\}$. We claim that $T_i$ is a subdivision of a phylogenetic tree $T'_i$ on some subset of *X*. By symmetry, we may assume without loss of generality that $i=1$. Since *N* has *m* roots and since, by Property (C1), no two roots of *N* are assigned the same color under $\sigma$, it follows that $T_1$ contains exactly one root of *N*. Moreover, and as a direct consequence of Property (C2), we have that $T_1$ is connected.

To see that $T_1$ is a tree, it suffices to show that $T_1$ does not contain a hybrid vertex of *N* and both its parents. Assume for contradiction that $T_1$ contains a hybrid vertex $h\in H(N)$ and its parents *u* and *v*. By definition of $\Gamma (N)$, $\{\gamma _u, \gamma _v\}$ is an edge of $\Gamma (N)$. Since $\sigma$ is a proper vertex coloring of $\Gamma '(N)$ it follows that $\sigma (\gamma _u) \ne \sigma (\gamma _v)$. This is a contradiction since $u,v\in V'$ and, therefore, $\sigma (\gamma _u)= s_1=\sigma (\gamma _v)$. Thus, $T_1$ must be a tree, as required.

Since, $\Gamma '(N)$ is an omni-extension of $\Gamma (N)$, the definition of $\sigma$ ensures that $L(T_1) \subseteq X$. It follows that $T_1$ is a subdivision of a phylogenetic tree $T'_1$ on a subset of *X*, as claimed.

Now let $F=\{T_1, \ldots , T_m\}$. Then, by construction, we have $L(T_i)\not =L(T_j)$, for all $1\le i<j\le m$. In view of our claim, every tree in *F* is a subdivision of a phylogenetic tree in the forest $F'=\{T'_1, \ldots , T'_m\}$ and $\bigcup _{T\in F'} L(T)=X$. Moreover, for all $i \in \{1, \ldots , m\}$, an arc (*u*, *v*) of *N* with $u,v \in V(T_i)$ is also an arc of $T_i$. It follows that *N* is obtained from *F* by adding arcs joining vertices from distinct trees of *F*. Thus, *N* is forest-based. That *N* is proper forest-based is a direct consequence of the construction of *F* from *N*. $\square$

Interestingly, Theorem 7 can be strengthened in case $m=2$ as follows.

### Theorem 8

Let *N* be a 2-rooted network on *X*. Then, *N* is proper forest-based if and only if $\Gamma (N)$ has a bipartite omni-extension.

### Proof

Suppose that *N* is proper forest-based 2-network with proper base forest $F=\{T_1,T_2\}$. Then, by Theorem 7, there exists an omni-extension $\Gamma '(N)$ that is 2-colorable.

**Fig. 9:**
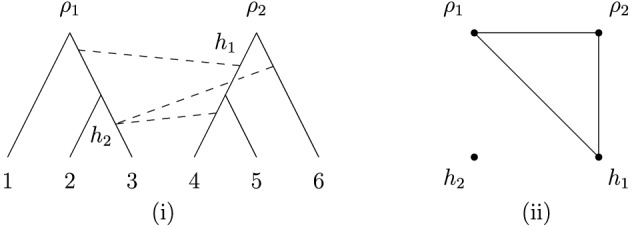
(i) A directed, acyclic graph *N* with two roots that is based on the forest indicated in solid edges. The three contact arcs are again dashed. (ii) The graph $\Gamma (N)$. Note that *N* is not semi-binary as $indeg_N(h_2)=3$

Conversely, suppose that there exists an omni-extension $\Gamma '(N)$ of $\Gamma (N)$ that is 2-colorable. Then, there exists a proper vertex coloring $\sigma :RH(N)\rightarrow \{s_1,s_2\}$, with $s_1 \ne s_2$. In view of Theorem 7, it suffices to show that $\sigma$ satisfies Properties (C1) and (C2).

Since *N* is connected, there must exist some hybrid vertex $h\in H(N)$ with parents $u',v'$ satisfying $\gamma _{u'}=\rho _1$ and $\gamma _{v'}=\rho _2$. So $\{\rho _1,\rho _2\}$ is an edge in $\Gamma (N)$, and therefore $\sigma (\rho _1)\not =\sigma (\rho _2)$ since $\sigma$ is a proper vertex coloring of $\Gamma '(N)$. Thus, Property (C1) holds.

To see that Property (C2) holds, consider the map $\psi =\psi _{\sigma }: V(N) \rightarrow \{\rho _1, \rho _2\}$ associated to $\sigma$ given by putting, for all $v\in V(N)$, $\psi (v)=\sigma (\gamma _v)$. Assume for contradiction that (C2) does not hold. Then, there must exist some $i\in \{1,2\}$, say $i=1$, and some vertex $g\in H(N)$ with $\sigma (\rho _1)=\sigma (g)$ such that every directed path from $\rho _1$ to *g* in *N* contains a vertex $r'\in H(N)$ for which $\sigma (r')\not =\sigma (\rho _1)$. Let *P* denote a directed path from $\rho _1$ to *g*. Without loss of generality, we may assume that $r'\in H(N)$ is a vertex on *P* such that, for every vertex $w\in V(N)$ on *P* strictly above $r'$, we have $\psi (w)=\sigma (\rho _1)$. Furthermore, we may assume without loss of generality that *g* is such that, for every $z\in V(N)$ on *P* that is strictly above *g* but below $r'$, we have $\psi (z)=\sigma (\rho _2)$.

Let $r\in V(N)$ denote the parent of *g* on *P* and let $g'\in V(N)$ denote the parent of $r'$ on *P*. Let $q\in V(N)$ denote the other parent of *g*. Then, by definition of *g*, it follows that $\{\gamma _{r},\gamma _q\}$ must be an edge in $\Gamma '(N)$. Since, by assumption, $\Gamma '(N)$ does not contain a cycle of length one (as otherwise $\Gamma '(N)$ would not be 2-colorable), it follows that $\gamma _{r}\not =\gamma _q$. Hence, $\sigma (\rho _2)=\psi (r)=\sigma (\gamma _{r})\not = \sigma (\gamma _q)$, and so $\sigma (\gamma _q)=\sigma (\rho _1)$ because $\sigma$ is a 2-coloring. If $\gamma _q$ is a vertex on *P* above $g'$, we obtain a contradiction, since the definition of *g* implies that we have found a directed path $P'$ from $\rho _1$ to *g* in *N* such that $\sigma (w)=\sigma (\rho _1)$ for all vertices $w\in H(N)$ contained in $P'$. By the choice of *g*, it follows that, $\gamma _q$ does not lie on *P*. Similar arguments as in the case of *g*, *r* and *q* imply that for one of the parents of $\gamma _q$ in *N*, *z* say, we also have $\sigma (\gamma _z)=\sigma (\rho _1)$. Repeating this argument, since *V*(*N*) is finite, we eventually obtain a directed path $P^*$ from $\rho _1$ to *g* in *N* such that $\sigma (\rho _1)=\sigma (h)$ holds for every hybrid vertex *h* on $P^*$, a contradiction. Thus, Property (C2) must hold. $\square$

Note that as Fig. 9i shows, the inclusion of the semi-binary requirement in the definition of a network is necessary for Theorem 8 to hold (since, extending relevant definitions for semi-binary networks in the obvious way to general networks *N* in which not every hybrid vertex must have indegree two, every omni-extension of $\Gamma (N)$ is a supergraph of $\Gamma (N)$, and $\Gamma (N)$ contains a cycle of length three). Also, the network *N* depicted in Fig. 7i shows that Theorem 8 need not hold for *m*-rooted networks with $m \ge 3$, since $\Gamma (N)$ is an omni-extension of itself because $\mathfrak {O}(N)=\emptyset$ and $\Gamma (N)$ is not bipartite.

## Universal Forest-Based Networks

**Fig. 10:**
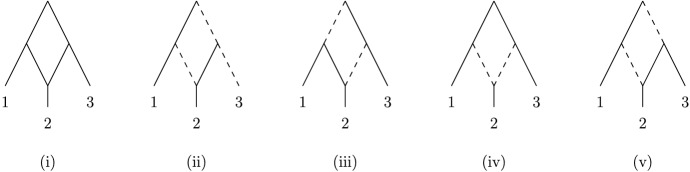
(i) A universal forest-based network on $X=\{1,2,3\}$. (ii)–(v) Embeddings of the four phylogenetic forests on *X* into the network in (i). In all cases, the dashed arcs represent contact arcs

It has been shown in Hayamizu (2016) and Zhang (2016) that there exist tree-based, binary phylogenetic networks *N* on *X* such every possible binary phylogenetic tree on *X* is a base-tree for *N*. Such binary networks are called *universal tree-based* networks. It is thus of interest to understand if there are binary universal forest-based networks (i.e., binary networks *N* such that every phylogenetic forest on *X* is a base forest for *N*). In case $|X| \le 3$, there always exists such a network (see Fig. 10 for $|X|=3$).

However, we now prove the following:

### Theorem 9

For all *X* with $|X|\ge 4$, there does not exist a universal forest-based network on *X*.

To prove this theorem, we begin with a useful observation.

### Lemma 5

**Fig. 11:**
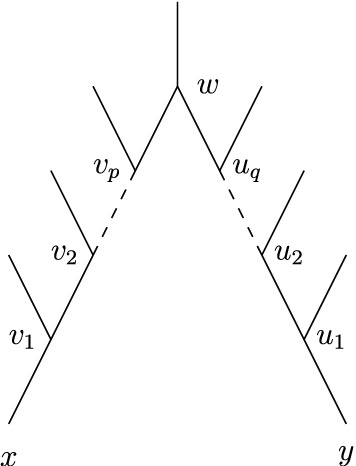
Forbidden configuration in a universal network. Note that *w* can also be a root vertex

Suppose that *U* is a universal forest-based network on *X*, $|X| \ge 4$. Then, for $x,y \in X$ distinct, and all $p,q \ge 0$, *U* does not contain the configuration pictured in Fig. 11 where $v_0=x$ and $q_0=y$.

### Proof

Assume for contradiction that for all $x,y\in X$ distinct there exists some $p,q\ge 0$ such that *U* contains the configuration in Fig. 11 where $v_0=x$ and $q_0=y$. Since *U* is universal forest-based and $|X|\ge 4$ there must exists a base forest *F* for *U* that has a component *T* which has two leaves $x,y \in X$ so that *x* and *y* are not contained in two arcs in *T* that have a common tail. In particular, *T* has at least 3 leaves. Let $F'$ be some embedding of *F* in *U* and let $T'$ be the corresponding embedding of *T*, which exists as *U* is universal.

Put $x=v_0$ and $y=q_0$. Let *w* denote a tree vertex or a root of *U* and, for all $1\le i\le p$ and all $1\le j\le q$, let $v_i$ and $u_j$ denote hybrid vertices of *U* such that $v_i$ is the parent of $v_{i-1}$ and $u_j$ is the parent of $u_{j-1}$ and *w* is the parent of $v_p$ and of $u_q$ (see the configuration depicted in Fig. 11). Then, since all $v_i$, $1\le i\le p$, and all $u_j$, $1\le j\le q$, are hybrid vertices of *U* it follows that $x=v_0,\dots ,v_p, y=u_0,\dots , u_q$ must all be contained in $T'$. But then at least one of $(w,v_p)$ and $(w,u_q)$ must be an arc in $T'$, otherwise *w* would be a leaf of some component in $F'$ and so $L(F)\not =L(U)$. Since *w* is a tree vertex or a root of *U* this implies that $w,v_p,u_q$ are vertices in $T'$. Thus, both arcs $(w,v_p)$ and $(w,u_q)$ must be arcs in $T'$. But this implies that *x* and *y* are contained in two arcs in *T* that have a common tail, a contradiction. $\square$

### Proof of Theorem 9

Assume for contradiction that there exists a universal forest-based network *U* on *X*. Let *w* be a root or tree vertex of *U* such that all non-leaf vertices below *w* are contained in *H*(*U*). Note that this configuration must exist since $|X|\ge 4$, and so there are at least two base forests on *X*. Let *u* and *v* be the children of *w*. By Lemma 5, there exists a unique leaf $x \in X$ of *U* such that *x* is a descendant of *w*. In particular, *x* is a descendant of both *u* and *v*. Now let *F* be a forest with two components, one of which is the tree $T_x$ whose sole vertex is *x* and the other is the phylogenetic tree *T* where *T* has leaf-set $X-\{x\}$. Let $F'$ be an embedding of *F* in *U* and $T'_x$ be the corresponding embedding of $T_x$ into *U* (which exists as *U* is universal). Note that $T'_x$ is a directed path ending at *x* and that *u* cannot be below *v* (or vice versa) since *U* is forest-based.

Since the directed paths from *u* to *x* and from *v* to *x* only contain hybrid vertices, $T_x'$ must contain both of these paths. But this is a contradiction, since the union of these paths must contain a hybrid vertex and both its parents.

$$\square $$

## Conclusion

In this paper, we have introduced the concept of forest-based networks and investigated some of their fundamental properties. We conclude by indicating some possible future directions of research for forest-based networks.

In Sect. 4, we studied the relationship between forest-based networks and other classes of networks. It could be interesting to investigate these relationships in more detail. For example, it is known that binary tree-child phylogenetic networks are precisely the tree-based networks such that every embedded phylogenetic tree is a base tree Semple (2016)—does a similar result hold for forest-based networks? In addition, in this paper we only considered properties of semi-binary networks. Which of our definitions and results extend to non-binary networks (i.e., networks that are not necessarily semi-binary)? Note that in Jetten and van Iersel (2018) properties of non-binary tree-based networks were considered, which might provide some useful leads to studying this question.

There are also several open algorithmic questions that could be investigated. For example, there are efficient algorithms for deciding whether a given phylogenetic network is tree-based or not, and if so to find a base-tree Francis and Steel (2015), Jetten and van Iersel (2018). Is there an efficient algorithms for deciding whether a given phylogenetic network is forest-based or not? In this regard, Theorem 7 might be useful as it could provide a useful link with coloring problems. It is also known to be NP-complete to decide whether or not a binary phylogenetic network is based on a given binary phylogenetic tree—does a similar algorithmic complexity result hold for forest-based networks?

Finally, it could be interesting to study related classes of networks. For example, *pedigrees*Steel and Hein (2006) are closely related to multiply rooted networks, and it is known that the two subgraphs of a pedigree induced by the bipartition of the pedigree into its male and female individuals are both forests (Semple and Steel 2003, Lemma 1.4.4). Are there interesting relationships between pedigrees and forest-based networks? Also, we could consider a generalization of tree-based *unrooted* phylogenetic networks which were first considered in Francis et al. (2018). In particular, an *unrooted forest-based network* *N* is an unrooted phylogenetic network on *X* (as defined in Francis et al. (2018)) that contains a spanning forest with leaf-set *X* such that no edge in *N* has both of its vertices in the same tree of the forest. What properties do such networks enjoy?

## Footnotes

1 Note that two equivalent phylogenetic networks are sometimes also called isomorphic.
